# Retraction Note: Development of attributes and attribute levels for a discrete choice experiment on patients’ and providers’ choice for antiretroviral therapy service in Northwest Ethiopia

**DOI:** 10.1186/s12981-023-00555-7

**Published:** 2023-08-24

**Authors:** Yihalem Abebe Belay, Mezgebu Yitayal, Asmamaw Atnafu, Fitalew Agimass Taye

**Affiliations:** 1 Department of Public Health, College of Health Sciences, Debre Markos University, Debre Markos, Ethiopia; 2 Department of Health Systems and Policy, Institute of Public Health, College of Medicine and Health Sciences, University of Gondar, Gondar, Ethiopia; 3 Department of Accounting, Finance, and Economics, Griffith University, Brisbane, Australia

**AIDS Research and Therapy (2023) 20:33**

10.1186/s12981-023-00531-1

The Editor-in-Chiefs have retracted this article because it contains material that substantially overlaps with the following articles (amongst others) [1, 2]. None of the authors have responded to any correspondence from the editor/publisher about this retraction.
